# Analysis of Several PLA_2_ mRNA in Human Meningiomas

**DOI:** 10.1155/2009/689430

**Published:** 2010-03-21

**Authors:** Yves Denizot, Rafael De Armas, Karine Durand, Sandrine Robert, Jean-Jacques Moreau, François Caire, Nicolas Weinbreck, François Labrousse

**Affiliations:** ^1^UMR CNRS 6101, Centre National de la Recherche Scientifique, Faculté de Médecine, Université de Limoges, 2 rue Dr. Marcland, 87025 Limoges, France; ^2^Service d'Anatomie Pathologique, CHU Dupuytren, 87045 Limoges, France; ^3^Service de Neurochirurgie, CHU Dupuytren, 87045 Limoges, France

## Abstract

In view of the important oncogenic action of phospholipase A_2_(PLA_2_) we investigated PLA_2_ transcripts in human meningiomas. Real-time PCR was used to investigate PLA_2_ transcripts in 26 human meningioma tumors. Results indicated that three Ca^2+^-dependent high molecular weight PLA_2_ (PLA_2_-IVA, PLA_2_-IVB, PLA_2_-IVC), one Ca^2+^-independent high molecular weight PLA_2_ (PLA_2_-VI) and five low molecular weight secreted forms of PLA_2_ (PLA_2_-IB, PLA_2_-IIA, PLA_2_-III, PLA_2_-V, and PLA_2_-XII) are expressed with PLA_2_-IVA, PLA_2_-IVB, PLA_2_-VI, and PLA_2_-XIIA as the major expressed forms. PLA_2_-IIE, PLA_2_-IIF, PLA_2_-IVD, and PLA_2_-XIIB are not detected. Plasma (PLA_2_-VIIA) and intracellular (PLA_2_-VIIB) platelet-activating factor acetylhydrolase transcripts are expressed in human meningiomas. However no difference was found for PLA_2_ transcript amounts in relation to the tumor grade, the subtype of meningiomas, the presence of inflammatory infiltrated cells, of an associated edema, mitosis, brain invasion, vascularisation or necrosis. In conclusion numerous genes encoding multiples forms of PLA_2_ are expressed in meningiomas where they might act on the phospholipid remodeling and on the local eicosanoid and/or cytokine networks.

## 1. Introduction

Meningiomas are the second most common primary intracranial tumor. Meningiomas present clinically by causing focal or generalized seizure disorders, focal neurological deficits or neuropsychological decline [1]. A large number of molecular and genetic pathways that are altered in brain tumor cells have been identified. Among them, a possible role for the eicosanoid cascade has been suggested in meningiomas [2]. Phospholipase A_2_ (PLA_2_) is the key enzyme involved in eicosanoid generation [3–5]. PLA_2_ catalyzes the hydrolysis of the *sn*-2 position of membrane glycerophospholipids to liberate the eicosanoid precursor arachidonic acid (AA) [3–5]. Four distinct families have been documented: low molecular weight secreted forms of PLA_2_ (sPLA_2_), Ca^2+^-dependent high molecular weight PLA_2_ (cPLA_2_), Ca^2+^-independent high molecular weight PLA_2_ (iPLA_2_); and the platelet-activating factor acetylhydrolase (PAF-AH). The sPLA_2_ family is implicated in several biological processes such as inflammation and host defense [3, 4]. Nine isoenzymes have been identified in human: PLA_2_-IB, PLA_2_-IIA, PLA_2_-IID, PLA_2_-IIE PLA_2_-IIF, PLA_2_-III, PLA_2_-V, PLA_2_-X, PLA_2_-XIIA, and PLA_2_-XIIB. In addition to their function in digestion of dietary phospholipids, host defense against bacteria and AA release from cellular phospholipids for eicosanoid synthesis, two classes of receptors (M and N) and several extracellular binding proteins have been identified indicating that sPLA_2_ might signal through receptor activation [3–5]. In human the cPLA_2_ family consists of four members, PLA_2_-IVA, PLA_2_-IVB, PLA_2_-IVC, and PLA_2_-IVD; PLA_2_-IVA being the central regulator of stimulus-coupled cellular AA release [3–5]. In human the iPLA_2_ group consists of seven members, iPLA_2_ (PLA_2_-VIA-1) currently being the best known member and playing major role in phospholipids remodeling and cancer [3, 5]. Beside its important place for eicosanoid generation, PLA_2_ is also the key enzyme for the generation of the pro-inflammatory lipid mediator PAF recently documented in human meningioma [6]. PAF-AH activity which hydrolysis PAF into the inactive PAF precursor, lyso-PAF is detected in meningioma [6]. However no results reported whether this enzymatic activity originated from PLA_2_-VIIA and/or PLA_2_-VIIB, the plasma PAF-AH and the intracellular PAF-AH forms, respectively. Currently the contribution of PLA_2_ in meningiomas is poorly documented despite the fact that PLA_2_ inhibition decreased the growth of cultured meningioma cells [7]. In view of the potentially important oncogenic action of the various PLA_2_ species, we investigated, at the mRNA levels, which of them were expressed in intracranial human meningiomas.

## 2. Materials and Methods

### 2.1. Patients

The procedure of the present study followed the rules edited by the French National Ethics. Ethics approval was obtained from the ethics committee of our hospital (CHU Dupuytren, Limoges, France). Twenty six patients who underwent surgery for intracranial meningiomas (from 1998 to 2004) were investigated. Tumors were from the Service d'Anatomie Pathologique of the CHU Dupuytren (France). After undergoing the routine hospital analysis, the excess of sample was kept at −80°C until use and this in accordance with the regulations in force in France. No written or oral consent was obtained because it is a study of samples already collected and referred to research prior the French bioethical low (2004). Thus, ethics committee explicitly approved the waiver of consent. Normal meninges were not available in our institution in light of our ethic committee law. The low amounts (10–15 mg) of available tumors only permitted investigations at the mRNA level. Tumors were classified according to the WHO criteria [8]. There were 16 grade I meningiomas including 8 transitional (2 man, 6 women, mean age 60 years), 3 meningothelial (1 man, 2 women, mean age 60 years), and 5 fibrous (5 women, mean age 59 years). Height tumors were grade II meningiomas: 7 atypical (6 men, 1 woman, mean age 58 years) and 1 chordoid meningiomas (1 man, 54 years). Two tumors were classified as anaplastic grade III meningiomas (2 men, mean age 54 years). Necrosis was assessed using morphological criteria defined by the WHO classification of meningiomas: “foci of spontaneous or geographic necrosis”. The chronic inflammatory infiltrate was mainly lymphoplasmocytic.

### 2.2. RNA Extraction

Total RNA was extracted using the “RNeasy Lipid Tissue mini kit” (Qiagen, Courtaboeuf, France) from 10–15 mg of tumor tissue. Before RNA extraction, tumor fragments were incubated with 14mm ceramic beads “Lysing matrix D” (Bertin Technologies, Montigny-Le-Bretonneux, France) in 1 mL QIAzol lysis reagent and homogenized at 5500 rpm during 2-fold 40 sec in the automated mixer Precellys (Bertin Technologies). Then homogenized samples were used for RNA purification according to the manufacturer's protocol. A DNase I digestion step was included for each extraction to avoid RNA contamination by genomic DNA. RNA integrity was checked by capillary electrophoresis on the Bioanalyzer 2100 (Agilent Technologies, Massy, France). Only RNA with an RNA Integrity Number (R.I.N) higher than 5.5 was used for reverse transcription. Total RNA concentration was determined by measuring absorbance at 260 nm with a spectrophotometre NanoDrop ND-1000 (Labtech, Paris, France). 

### 2.3. Reverse Transcription

Total RNA was reverse transcribed in single strand cDNA using random hexamers and as described in the protocol of the “SuperScript III First-Strand Synthesis System for RT-PCR” (Invitrogen, Cergy-Pontoise, France). Briefly, 1 *μ*g total RNA was incubated with 200 U M-MLV reverse transcriptase in the presence of 0.5 mM dNTPs, 50 ng random hexamers, 5 mM MgCl_2_, 10 mM dithiotreitol, and 40 U RNase inhibitor, in a final volume of 20 *μ*L 1X RT buffer. Reverse transcription was performed as follows in a thermocycler Gold 9700 (Applied Biosystem): denaturation during 5 min at 65°C and chilling 1 min on ice, hybridization during 10 min at 25°C followed by cDNA synthesis during 50 min at 50°C; enzyme was inactivated by a 5 min incubation at 85°C and chilling on ice; finally the destruction of the RNA portion of the RNA:cDNA hybrids was performed by 2 U RNase H during 20 min at 37°C. Reactions were frozen at −20°C until quantitative real-time PCR realisation.

### 2.4. Real-Time PCR Analysis

**Figure 1:**
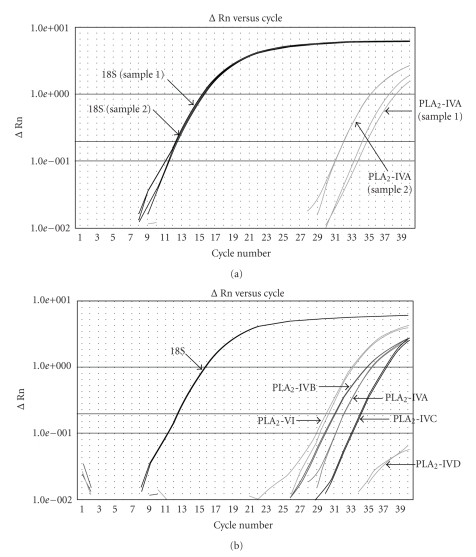
Q-PCR analysis of PLA_2_ transcripts in human meningioma tumors. (a) Representative tracing of 18S and PLA_2_-IVA of tumors from 2 different patients (duplicate samples). (b) Detection (in duplicate) of 18S, PLA_2_-IVA, PLA_2_-IVB, PLA_2_-IVC, PLA_2_-IVD, and PLA_2_-VI in a meningioma tumor.

PLA_2_-IB, PLA_2_-IIA, PLA_2_-IID, PLA_2_-IIE, PLA_2_-IIF, PLA_2_-III, PLA_2_-IVA, PLA_2_-IVB, PLA_2_-IVC, PLA_2_-IVD, PLA_2_-V, PLA_2_-VI, PLA_2_-X, PLA_2_-XIIA, PLA_2_-XIIB, PLA_2_-VIIA (plasma PAF-AH), PLA_2_-VIIB (intracellular PAF-AH), and PLA_2_R transcripts were analyzed using real-time polymerase chain reaction (PCR). PCR was performed in duplicate by using TaqMan assay reagents (Applied Biosystems, Foster City, CA) [9, 10]. Product references were the following: PLA_2_-IB: Hs00386701-m1; PLA_2_-IIA: Hs00179898-m1; PLA_2_-IID: Hs00173860-m1 PLA_2_-IIE: Hs00173897-m1; PLA_2_-IIF: Hs00224482-m1; PLA_2_-III: Hs00210447-m1; PLA_2_-IVA: Hs00233352-m1; PLA_2_-IVB: Hs00979952-m1; PLA_2_-IVC: Hs00234345-m1; PLA_2_-IVD: Hs00603557-m1; PLA_2_-V: Hs00173472-m1; PLA_2_-VI: Hs001/85926-m1; PLA_2_-X: Hs00358567-m1; PLA_2_-XIIA: Hs00830106-s1; PLA_2_-XIIB: Hs00261432-m1; PLA_2_-VIIA: Hs00968593-m1; PLA_2_-VIIB: Hs01042135-m1; PLA_2_R: Hs00234853-m1. Real-time PCR were performed following the recommendations of the manufacturer in a final volume of 20 *μ*l with 10 *μ*l of 2X Universal PCR Master Mix, 20 ng of cDNA in a volume of 9 *μ*l (the amount of cDNA is an equivalent based on the initial amount of RNA used for the RT reaction and 1 *μ*l of a 20X TaqMan gene expression specific probe. PCR parameters were the following: 95°C for 10 min and forty cycles of 95°C/15 sec and 60°C/60 sec. Amplification products were analyzed on an ABI Prism 7000 system (Applied Biosystems) [9, 10]. Gene expression levels were normalized to 18S RNA (product reference: Hs99999901-s1) according to the manufacturer's recommendation. Amounts of various transcripts were compared to sample with the lowest level of transcripts (a patient who was arbitrary quoted 1). The relative quantification of gene expression was performed using the comparative *C*_*T*_ method (∆∆*C*_*T*_) (Figure 1(a)). Experiments were made in duplicate. Mean *C*_*T*_ values were used in the ∆∆*C*_*T*_ calculation by using the “relative quantitation calculation and analysis software for Applied Biosystems real-time PCR systems”. NonRT controls (only with RNA) and blank RT controls (RT without RNA) were run to make sure the amplifications were specifics.

### 2.5. Data Analysis

Significance was assessed by using the Kruskal-Wallis test followed by a Mann-Whitney *U*-test.

## 3. Results and Discussion

**Figure 2:**
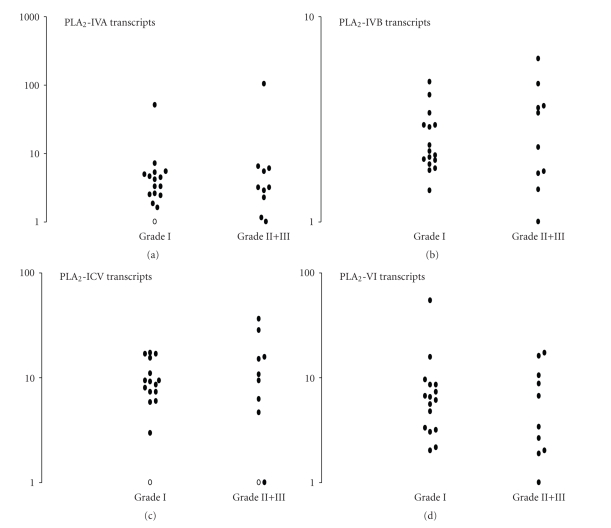
Cytosolic PLA_2_ transcripts in human meningiomas. Sixteen-grade I and eleven-grade II+III meningiomas were investigated. Gene expression levels were normalized to 18S RNA. Amounts of transcripts were compared to sample with the lowest level of transcripts (a patient who was arbitrary quoted 1). (∘) indicates patients with no detectable transcripts. No significant differences were documented between groups.

In a first set of experiments we investigated if mRNAs derived from the five intracellular PLA_2_ genes (four cPLA_2_ and iPLA_2_) were detected in meningiomas. As shown in Figure 1(b), mRNAs derived from four of these five cloned PLA_2_ genes are detected. Mean *C*_*T*_ values are reported in Table 1. PLA_2_-IVD transcripts were not present at detectable levels. In contrast, PLA_2_-IVA, PLA_2_-IVB, PLA_2_-IVC, and PLA_2_-VI were detected in 96% (25/26), 100% (26/26), 92% (24/26), and 100% (26/26) of tumors, respectively (Figure 2). These results confirm a previous study highlighting PLA_2_ activity in 100% of human meningiomas [6]. No difference (*P* > .05, Mann Whitney *U*-test) was found for PLA_2_ transcript amounts in relation to the tumor grade (Figure 1), nor the subtype of meningiomas, the presence of inflammatory infiltrated cells, of an associated edema, mitosis, brain invasion, vascularisation or necrosis (data not shown). The analysis of twenty six patients indicated the following rank of magnitude in human meningiomas: PLA_2_-VI = PLA_2_-IVB > PLA_2_-IVA > PLA_2_-IVC (Table 1). PLA_2_-IVB and PLA_2_-IVC had little specificity for the *sn*-2 fatty acid as compared with PLA_2_-IVA which preferentially hydrolyses phospholipids containing AA at the *sn*-2 position [3–5]. PLA_2_-VI was originally reported to mediate phospholipid remodeling and, thus, to act as a housekeeping protein without significant role in cell growth [3, 4]. However several recent studies have demonstrated that PLA_2_-VI exhibited roles in cell regulation, growth, and death. Especially, one mechanism by which PLA_2_-VI mediates cell growth involves regulation of AA release, p53, and MAPK activation [11]. Of interest, involvements of p53 and MAPK kinase have been recently reported in the pathology of human meningiomas [12, 13]. A role for PLA_2_-VI may, thus, be suggested in meningioma tumor growth. Together, these observations might suggest PLA_2_-VI as a novel and interesting target for drug development for meningioma therapy. However, given the ubiquitous expression of PLA_2_-VI and its role in glycerophospholipid metabolism, drug strategies targeting PLA_2_-VI must exhibit selectivity to avoid undesired side effects. 

**Figure 3:**
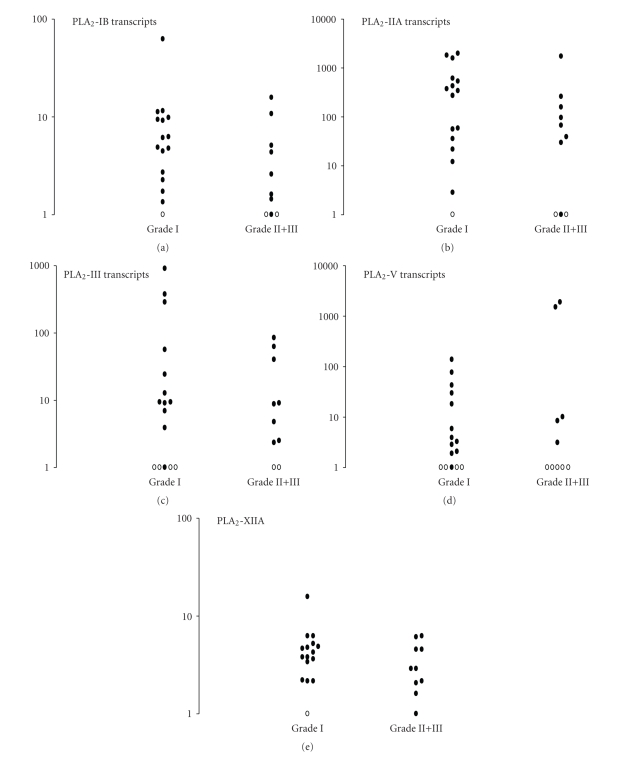
Secreted PLA_2_ transcripts in human meningiomas. Same legend as in Figure 2. (∘) indicates patients with no detectable transcripts. No significant differences were documented between groups.

In a second set of experiments we investigated if mRNAs derived from the nine sPLA_2_ genes (i.e., PLA_2_-IB, PLA_2_-IIA, PLA_2_-IID, PLA_2_-IIE, PLA_2_-IIF, PLA_2_-III, PLA_2_-V, PLA_2_-X, PLA_2_-XIIA, and PLA_2_-XIIB) were detected in human meningiomas. PLA_2_-IIE, PLA_2_-IIF, and PLA_2_-XIIB transcripts were not present at detectable levels in tumors while PLA_2_-IID and PLA_2_-X transcripts were detected in only a few number (4/26, 15%) of them. In contrast, PLA_2_-IB, PLA_2_-IIA, PLA_2_-III, PLA_2_-V and PLA_2_-XIIA were detected in 88% (23/26), 88% (23/26), 77% (20/26), 65% (17/26), and 96% (25/26) of tumors, respectively (Figure 3). Mean C_T_ values are reported in Table 1. Results indicated the following rank of magnitude for sPLA_2_ transcripts in meningiomas: PLA_2_-XIIA > PLA_2_-IIA > PLA_2_-IB = PLA_2_-V = PLA_2_-III = PLA_2_-IID > PLA_2_-X. No difference (*P* > .05, Mann Whitney *U*-test) was found for sPLA_2_ transcript amounts in relation to the tumor grade (Figure 3), nor the subtype of meningiomas, the presence of inflammatory infiltrated cells, of an associated edema, mitosis, brain invasion, vascularisation or necrosis (data not shown). PLA_2_-XIIA, PLA_2_-IIA, and PLA_2_-IB might be implicated in meningioma growth. The physiologic roles of PLA_2_-XIIA remain an open question. Whether PLA_2_-XIIA exhibits a weak AA catalytic activity [14], a potential role for this enzyme is suggested in membrane fusion or cell division [15]. PLA_2_-XIIA was reported to inhibit bone morphogenetic protein (BMP) through the loss of activated Smad1/4 complexes [16]; a result of importance since BMP inhibits the tumorigenic potential of human glioblastomas by triggering the Smad signaling cascade [17]. PLA_2_-IB expression is mainly neuronal in human brain [18]. Apart from its lipolytic and pro-inflammatory activities, PLA_2_-IB acts as receptor ligand to induce cell signaling and subsequent activation of cPLA_2_, thus indirectly contributing to AA production [19]. However the role of PLA_2_-IB as a ligand for the PLA_2_R is still controversial. PLA_2_-IIA elicits a mitogenic response and activates AA metabolism in astrocytoma cells [20], is critical for neuronal death via reactive oxygen species [21] and plays a role in cellular senescence [22]. Finally, studies have suggested the role of PLA_2_-IIA, PLA_2_-III, and PLA_2_-V as potential prognostic markers in colorectal adenocarcinomas and prostate cancer [23, 24]. The data reported in Figure 3 suggest that levels of PLA_2_-IIA, PLA_2_-III, and PLA_2_-V transcripts greatly varied between patients (see the Log scale). Would it be possible that their levels were related to patient outcomes in meningioma tumors? Clearly investigation of a larger number of patients would be of interest to test this hypothesis.

**Figure 4:**
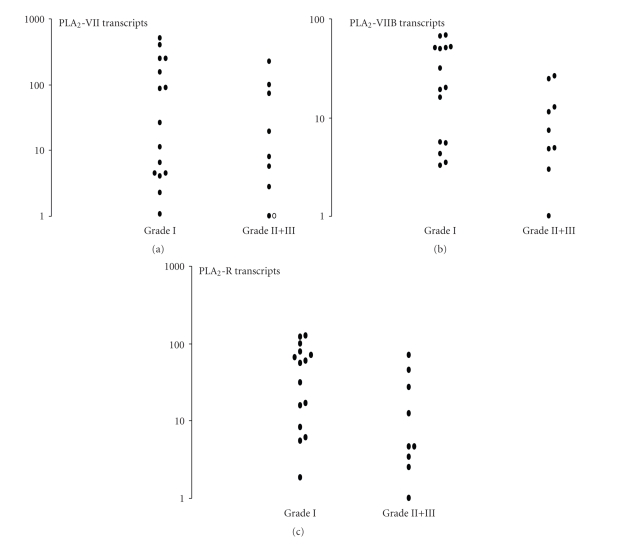
PAF-AH and PLA_2_R transcripts in human meningiomas. Upper panel: plasma PAF-AH (PLA_2_-VIIA) and intracellular PAF-AH (PLA_2_-VIIB) in meningioma tumors. Fifteen-grade I and nine-grade II+III meningiomas were investigated. (∘) indicates patients with no detectable transcript. No significant differences were documented between groups. Lower panel: PLA_2_R transcripts in fifteen-grade I and nine-grade II+III meningiomas.

In a third set of experiments we investigated PAF-AH enzymes that constitute another PLA_2_ subfamily. As shown in Figure 4 (upper panel), PLA_2_-VIIA (the plasma PAF-AH form) and PLA_2_-VIIB (the intracellular PAF-AH form) were present in 100% (23/23) and 95% (22/23) meningioma tumors. Mean C_T_ values are reported in Table 1. No difference (*P* > .05, Mann Whitney *U*-test) was found for PAF-AH transcript amounts in relation to the tumor grade (Figure 4(a)), nor to other clinical data (data not shown). The present results confirm a previous study reporting PAF-AH enzymatic activity in meningioma homogenates [6]. The PAF-AH family exhibits unique substrate specificity toward PAF and oxidized phospholipids. Degradation of these bioactive phospholipids by PAF-AH may lead to the termination of inflammatory reaction. Its presence in human meningioma is consistent with the presence of PAF in meningioma tumors [6, 25].

Finally in a fourth set of experiments we focused our attention on PLA_2_R transcripts in human meningioma. As shown in Figure 4 (lower panel), PLA_2_R transcripts were detected in 100% (23/23) meningioma tumors but without significant link with the tumor grade. The PLA_2_R can act as a ligand for several sPLA_2_ thus mediating a variety of biological responses (such as cell proliferation, cell migration, hormone release, lipid mediator production and cytokine production). In turn, PLA_2_R can also play a negative role in sPLA_2_ functions by downregulating their exaggerated reactions as PLA_2_R is involved in the degradation/internalization of sPLA_2_ [26]. Particularly, PLA_2_R deficient mice exhibit resistance to endotoxic shock [27] and knockdown of the PLA_2_R prevents the onset of replicative senescence and diminishes stress-induced senescence [28]. Finally PLA_2_R was found to be upregulated in dermatofibrosarcoma [29].

In conclusion, numerous genes encoding multiples forms of cPLA_2_, sPLA_2_, and PAF-AH are expressed (at the mRNA level) in human meningiomas where they might act on tumor growth not only by acting on phospholipid remodeling but also by altering the local eicosanoid and/or cytokine networks. It is of evidence that immunhistochemistry would be of importance to confirm the relative expression of the different PLA_2_ forms in human meningioma tumors. The discovery of specific receptors that bind sPLA_2_ strongly indicate that these enzymes can exert various biological responses via binding to a receptor, in addition to their enzymatic activity. Of interest meningioma tumors expressed PLA_2_R transcripts. Further studies are clearly needed to elucidate the contributions of sPLA_2_, cPLA_2_, iPLA_2_, and PAF-AH in meningioma and to determine their possible relevance in the targeting of new therapeutic interventions.

## Figures and Tables

**Table 1:** *C*_*T*_ values obtained during real-time PCR analysis.

	Mean ± SEM *C*_*T*_ (made on detectable samples)	Number of samples with a *C*_*T*_ > 40 (nondetectable samples)
18S	12.27 ± 0.18	0
PLA_2_-IB	35.35 ± 0.29	3
PLA_2_-IIA	33.69 ± 0.74	3
PLA_2_-IID	34.91 ± 0.81	22
PLA_2_-IIE	nd	26
PLA_2_-IIF	nd	26
PLA_2_-III	35.28 ± 0.50	6
PLA_2_-IVA	32.3 ± 0.27	1
PLA_2_-IVB	30.57 ± 0.33	0
PLA_2_-IVC	33.9 ± 0.35	2
PLA_2_-IVD	nd	26
PLA_2_-V	35.81 ± 0.61	9
PLA_2_-VI	30.78 ± 0.30	0
PLA_2_-VIIA	33.19 ± 0.48	0
PLA_2_-VIIB	30.25 ± 1.21	1
PLA_2_-X	37.93 ± 0.26	22
PLA_2_-XIIA	32.86 ± 0.33	1
PLA_2_-XIIB	nd	26

Results are reported as mean ± SEM of 26 experiments excepted for PLA_2_-VIIA and PLA_2_-VIIB were 23 samples were analysed. nd: not detectable *C*_*T*_.

## Abbreviations

PLA_2_: phospholipase A_2_
sPLA_2_: secreted phospholipase A_2_
cPLA_2_: cytosolic phospholipase A_2_
iPLA_2_: calcium independent phospholipase A_2_
PCR: polymerase chain reaction.
